# eTOX ALLIES: an automated pipeLine for linear interaction energy-based simulations

**DOI:** 10.1186/s13321-017-0243-x

**Published:** 2017-11-21

**Authors:** Luigi Capoferri, Marc van Dijk, Ariën S. Rustenburg, Tsjerk A. Wassenaar, Derk P. Kooi, Eko A. Rifai, Nico P. E. Vermeulen, Daan P. Geerke

**Affiliations:** 1 AIMMS Division of Molecular Toxicology, Department of Chemistry and Pharmaceutical Sciences, Vrije Universiteit Amsterdam, De Boelelaan 1108, 1081 HZ Amsterdam, The Netherlands; 2 Present Address: Computational Biology Program, Memorial Sloan Kettering Cancer Center, New York, NY 10065 USA; 3 Present Address: Groningen Biomolecular Sciences and Biotechnology Institute and Zernike Institute for Advanced Materials, University of Groningen, 9747 AG Groningen, The Netherlands

**Keywords:** Binding affinity prediction, Free energy calculation, Linear interaction energy, Drug design, Computational toxicology

## Abstract

**Background:**

Computational methods to predict binding affinities of small ligands toward relevant biological (off-)targets are helpful in prioritizing the screening and synthesis of new drug candidates, thereby speeding up the drug discovery process. However, use of ligand-based approaches can lead to erroneous predictions when structural and dynamic features of the target substantially affect ligand binding. Free energy methods for affinity computation can include steric and electrostatic protein–ligand interactions, solvent effects, and thermal fluctuations, but often they are computationally demanding and require a high level of supervision. As a result their application is typically limited to the screening of small sets of compounds by experts in molecular modeling.

**Results:**

We have developed *eTOX ALLIES*, an open source framework that allows the automated prediction of ligand-binding free energies requiring the ligand structure as only input. *eTOX ALLIES* is based on the linear interaction energy approach, an efficient end-point free energy method derived from Free Energy Perturbation theory. Upon submission of a ligand or dataset of compounds, the tool performs the multiple steps required for binding free-energy prediction (docking, ligand topology creation, molecular dynamics simulations, data analysis), making use of external open source software where necessary. Moreover, functionalities are also available to enable and assist the creation and calibration of new models. In addition, a web graphical user interface has been developed to allow use of free-energy based models to users that are not an expert in molecular modeling.

**Conclusions:**

Because of the user-friendliness, efficiency and free-software licensing, *eTOX ALLIES* represents a novel extension of the toolbox for computational chemists, pharmaceutical scientists and toxicologists, who are interested in fast affinity predictions of small molecules toward biological (off-)targets for which protein flexibility, solvent and binding site interactions directly affect the strength of ligand-protein binding.

**Electronic supplementary material:**

The online version of this article (10.1186/s13321-017-0243-x) contains supplementary material, which is available to authorized users.

## Background

Interactions between ligands and proteins represent an important step in many life-regulating signal-transmission processes. Upon molecular recognition a ligand can modulate protein function, thereby enhancing, inhibiting or modulating its activity. The magnitude of the effect will depend on the strength or *affinity* of ligand-protein binding. Furthermore, in a complex biological system in which multiple interacting partners are present, the effect exerted by a ligand also depends on binding *selectivity*, i.e., the relative binding affinity toward a target when compared to other proteins. Therefore, a common goal in drug design, discovery and safety pharmacology is to obtain a compound with both high affinity for the protein of interest (target) and with high selectivity against other proteins for which activity modulation could lead to unwanted and possibly toxic events (off-targets) [1, 2]. In this light, computational approaches that are able to accurately predict the affinity of potential drug candidates toward targets and off-targets can help in identifying and optimizing new biologically active compounds [3, 4]. Such computational methods can be divided in ligand-based and protein-structure based approaches. In the first group, which mainly comprises quantitative structure-activity relationships (QSAR) models, statistical methods are applied to identify quantitative patterns between the structure of a chemical compound (represented as a series of molecular descriptors) and a specific biological property [5]. The fundamental assumption in QSAR is that compounds with similar structure share analogue biological properties, therefore structural information about the interacting partner is usually neglected. Furthermore, measures of similarity among structures can vary a lot depending of the metric in which it is estimated [6, 7]. Protein-structure based methods combine structural features of both the ligand and the interacting biological molecule to predict the binding affinity of the compound, usually quantified as the free energy of binding ($\Delta G_{bind}$) [3]. In this regard, empirical scoring functions have been developed to provide a fast estimation of $\Delta G_{bind}$ during screening of large dataset of compounds [8]. However, the high efficiency of this approach comes at the expense of its accuracy, which is often low for quantitative $\Delta G_{bind}$ prediction [9]. On the other hand, more rigorous statistical-mechanics based approaches such as free-energy perturbation (FEP) [10] and thermodynamic integration (TI) [11] can provide accurate $\Delta G_{bind}$ predictions that include thermal conformational sampling by means of e.g. molecular dynamics (MD) simulation [12, 13]. However, these calculations typically require numerous and extensive simulations involving non-physical states of the system of interest, making them computationally demanding and therefore not yet suitable for screening of large sets of compounds. As an alternative, approximations to these techniques led to the development of methods in which only the physical protein-bound and unbound states of the ligand are evaluated in simulation, substantially reducing computational costs while still including thermal effects on binding [14]. Among these methods, Linear Interaction Energy (LIE) theory is an empirical approach in which binding free energies are predicted by considering only the intermolecular interactions between the ligand and its environment in both end states [15]. Although QSAR methods still represent the most commonly used approach to predict ligand-binding affinities in applied settings, structure-based models are becoming more attractive due to the increased availability of three-dimensional structures of molecular (off-)targets and of computational resources. However, extensive application of methods to calculate $\Delta G_{bind}$ from simulation is also hindered by the high degree of supervision and technical knowledge that is typically required. To overcome these issues, we as well as others have in the past years developed extensions of the relatively efficient LIE method that open up possibilities for fully automated affinity prediction.

According to LIE, $\Delta G_{bind}$ can be computed from the difference in the ensemble Lennard–Jones $\big \langle V_{lig-surr}^{LJ}\big \rangle$ and electrostatic $\big \langle V_{lig-surr}^{Coul}\big \rangle$ interaction energies between the ligand and its environment as obtained from MD simulations of the ligand in complex with the protein (*protein*) and free in solvent (*water*) [15, 16]:

1 $$\begin{aligned} \begin{aligned} \Delta G_{bind}&= \alpha \ \Big ( \big \langle V_{lig-surr}^{LJ}\big \rangle _{protein} - \big \langle V_{lig-surr}^{LJ}\big \rangle _{water}\Big ) \\&\quad +\, \beta \ \Big ( \big \langle V_{lig-surr}^{Coul}\big \rangle _{protein} - \big \langle V_{lig-surr}^{Coul}\big \rangle _{water}\Big ) \\&= \alpha \ \Delta V ^{LJ} + \beta \ \Delta V ^{Coul} \end{aligned} \end{aligned}$$

$\alpha$ and $\beta$ in Eq. (1) are empirical parameters that can be fitted using a training set of ligands with known binding affinity toward a specific protein. After calibration, the LIE model can be used to predict $\Delta G_{bind}$ for query compounds with unknown affinity [16].

Similar to other free energy methods, predicted values may well depend on the conformation of the ligand-protein complex that is chosen by the user to start MD from [17]. As a remedy, Stjernschantz and Oostenbrink [18] proposed an extension to the LIE method in which the contributes obtained in simulations starting from different conformations of the ligand-protein complex could be included within a single model . $\Delta G_{bind}$ of a ligand can be expressed as averaged sum over the independent simulations *i*,

2 $$\begin{aligned} \Delta G_{bind} = \alpha \sum _{i}{W_{i}\ \Delta V^{LJ}} + \beta \sum _{i}{W_{i}\ \Delta V^{Coul}} \end{aligned}$$

where the relative contribute $W_{i}$ of *i* can be derived from [19]

3 $$\begin{aligned} W_{i}= \frac{e^{ -\frac{\Delta G_{bind,i}}{k_{B}T}}}{\sum _{i}{ e^{ -\frac{\Delta G_{bind,i}}{k_{B}T}}}} \end{aligned}$$

with $k_{B}$ Boltzmann’s constant and *T* the temperature.

Considering that the $W_{i}$’s depend on $\alpha$ and $\beta$ (and vice versa), Stjernschantz and Oostenbrink proposed a scheme in which $\alpha$, $\beta$ and $W_i$’s could be obtained in an iterative way during model fitting [18]. Beside leading to more accurate models due to inclusion of multiple (separate) parts of conformational space of the ligand-protein complex [18, 20], this scheme made LIE models independent from the *a priori* selection of ligand-binding poses, laying the basis for fully unsupervised LIE free energy predictions.

Due to their complexity, fully automated predictions require the implementation and/or integration of generic procedures for (1) generation of ligand-protein conformations to start MD from, (2) preparation of the force-field topology of the system, (3) running and (4) analysis of the MD simulations, and finally (5) the actual LIE-based $\Delta G_{bind}$ estimation [21].

Partially automated workflows have been developed previously to facilitate the set up and execution of LIE-based binding free energy calculations for protein–ligand complexes [21–23]. However, they still require manual intervention (e.g. for the preparation of topologies or ligand-protein complex coordinates). Furthermore the use of commercial propriety software or textual user interfaces can limit their usage to a restricted group of users.

To overcome these issues, we present here *eTOX ALLIES* (Automated pipeLine for Linear Interaction Energy-based Simulations), which allows for the calibration and (in-house) use of LIE models for $\Delta G_{bind}$ estimations in a fully automated way. Requiring the chemical structure of ligand(s) as input (submitted as *Structure Data Format* (*SDF*) file), *in silico* screening of compounds can be performed: docking into the (off-)target is performed for each ligand and relevant binding poses are selected from a statistical-geometric analysis of the docking results; subsequently, the interaction energies of representative binding poses are evaluated during MD, and results are collected and employed to compute $\Delta G_{bind}$’s. In addition, an (automated) protocol to estimate the reliability of each prediction has been implemented, according to our recently proposed approach [24]. The pipeline employs open source third-party software only, making no restriction for its use in both academic and private environment. Furthermore, a web graphical user interface (GUI) has been developed that allows for use and creation of new models in a user-friendly manner, making the tool accessible also to users that are not an expert in modeling.

Overall, *eTOX ALLIES* represents a new computational tool for pharmaceutical scientists, toxicologist and modelers, both from academia and industry, who are interested in predicting binding affinities toward (off-)targets for which structural features of the binding site and/or thermal conformational effects can significantly affect the ligand-binding process.

## Implementation

### Software architecture

*eTOX ALLIES* has been designed to provide a complete pipeline for creation and use of LIE-based models for $\Delta G_{bind}$ calculation of small molecules toward (off-)targets with known three-dimensional structure. The code has been written in *Python 2.7* [25] and makes use of external open source softwares. Molecular (ligand) structures are handled by *Open Babel* libraries [26, 27], while *SciPy* [28] and *scikit-learn* modules are employed for statistical analysis [29]. *ParaDockS* [30] and *GROMACS* [31] are adopted as docking and MD engines, respectively. A web interface for easy handling of job and model submission and management has been built adopting the python framework *Flask* [32], and using *Open Babel* [27] and *matplotlib* [33] modules for structure and plot representations.

**Fig. 1:**
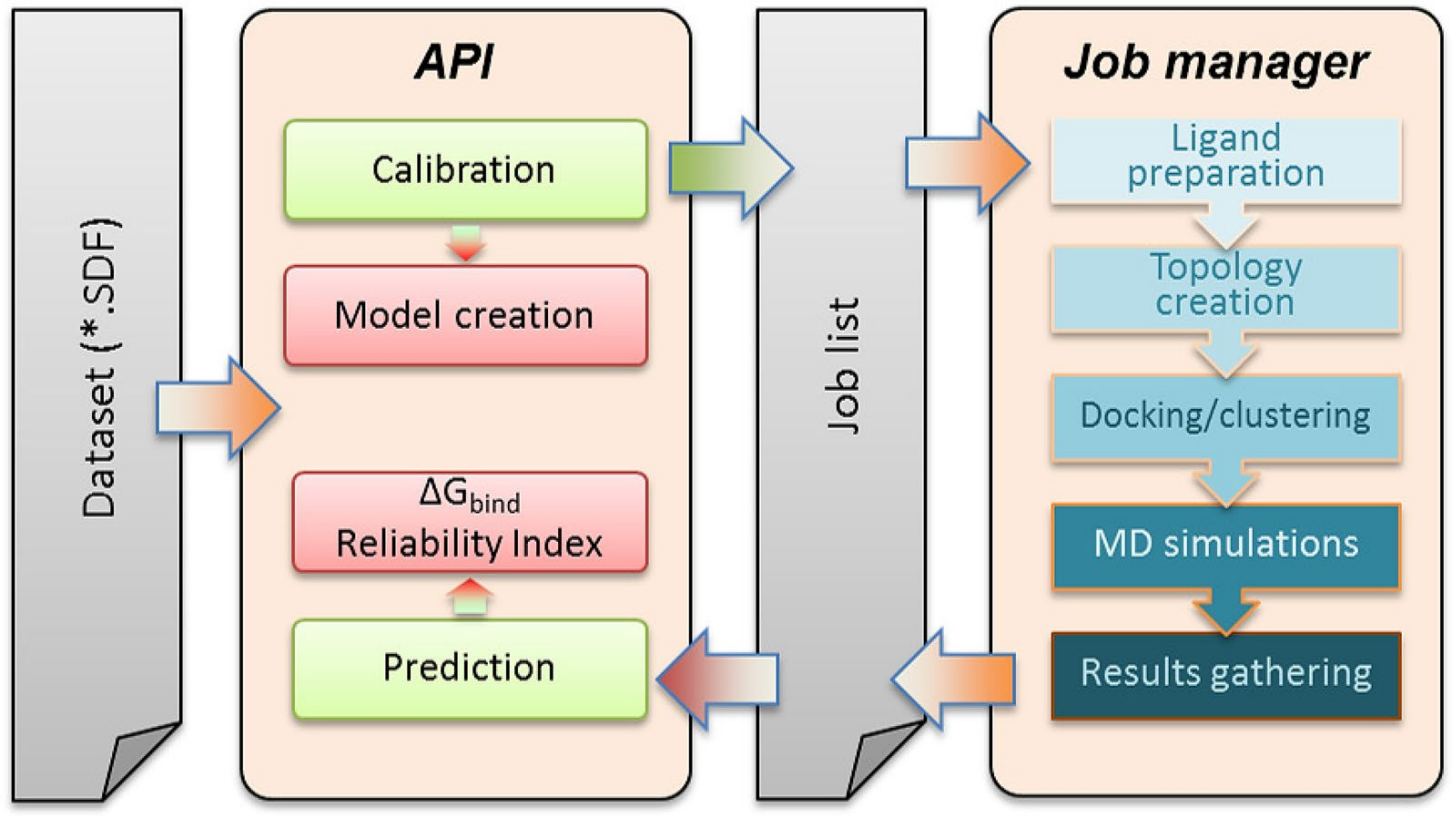
Architecture of eTOX ALLIES. $\Delta G_{bind}$ prediction or calibration (training) of a new model is initiated by submitting a dataset of compounds through the API. For each query or training compound, evaluation of ligand–protein interaction energies is requested using model-specific settings. The job manager takes care of the steps required to obtain interaction energies for each compound and to expose them to the API when completed. Interaction energies are subsequently used for model calibration or prediction, and as part of the evaluation of the reliability index for predictions

The software is organized in two main parts (Fig. 1): the *Job Manager* performs the steps required to obtain the descriptors used in the model for each ligand (from ligand-structure preparation and optimization, to gathering of interaction energies from MD simulations), while the *Application Programming Interface (API)* allows for easy submission of new-model calibrations or screening of query compounds.

### The Job Manager

The *Job Manager* runs in background and is responsible for obtaining ligand–protein interaction energies ($\big \langle V_{lig-surr}\big \rangle$ terms in Eq. (1)). For each ligand, the process includes: (1) ligand preparation, (2) ligand topology creation and structure optimization, (3) identification of representative ligand-protein complex conformations from docking and clustering, (4) MD simulations, and (5) postprocessing and gathering of interaction energies. External open source software is employed for execution of some of these steps (i.e., ligand-topology creation, docking, MD simulations) and is executed within the main framework using the *subprocess* module as interface.

#### 1. Ligand preparation

An *SDF* file is used to submit query or training compounds. Structural information contained in these files can be incomplete or not appropriate (e.g. improper protonation state, two-dimensional coordinates, etc.), therefore a preliminary structure optimization is performed.

During this first step, preprocessing of the ligand is performed using *Open Babel* [27], consisting of generation of 3D coordinates (in case 2D coordinates are provided only) and neutralization or protonation according to a pH of 7.4 (depending on the model settings).

#### 2. Ligand topology creation and structure optimization

The structure of the ligand(s) must be processed to provide suitable 3D coordinates as input for molecular docking, and to generate the force-field potential parameters that will describe bonded and nonbonded interactions involving the ligand during MD (referred to as topology).

After preprocessing, the optimized structure of and atomic charges for the ligand are obtained according to the AM1-BCC method [34] and the topology of the ligand is generated according to the General Amber Force Field (GAFF) [35]. These tasks are performed by the *sqm* and *antechamber* packages provided in *AmberTools15* [36]. The optimized geometry of the ligand is employed as input for subsequent docking, while the topology obtained is converted to *GROMACS* [31] format using *ACPYPE* [37].

#### 3. Identification of representative ligand-protein complex conformations

The representative ligand-protein complex conformations that will be used as starting structures for the MD simulations are obtained through clustering of binding poses obtained during molecular docking:

The optimized ligand structure is initially rotated by ± 90 degree in the *x*, *y* or *z* direction [38]. The ligand is subsequently docked into the protein binding site (using settings that can be defined using the API during model calibration), and maximally 50 poses with mutual RMSD of 2.0 Å are retained for each of the six rotated configurations. A principal component analysis (PCA) of the docked poses (represented as heavy-atom coordinates) is performed to reduce the number of variables, cf. [24]. The components explaining more than 5% of the initial variance are retained, and the corresponding scores are used in subsequent *k*-means clustering [39]. An increasing number of clusters is considered in case it would explain at least 5% more of the variance in the score space. The medoids of the clusters obtained are considered as representative binding poses and are used as starting configurations for the MD simulations of the ligand-protein complex.

#### 4. MD simulations

MD simulations allow the inclusion of solvent and thermal fluctuations during the evaluation of ligand–protein interaction energies.

Simulations are carried out using the *GROMACS 4.5* package [31]. An optimized version of the bash script used on the WeNMR GRID web portal [40] is adopted here to facilitate the process. Each protein-ligand complex is solvated in a dodecahedral box filled with TIP3P water [41], and $\hbox {Na}^{+}$ or $\hbox {Cl}^{-}$ ions are added to neutralize the charge of the protein. After energy minimization, the system is gradually heated to 300 K in (protein and ligand) heavy-atom restrained *NVT* simulations of 10 ps simulations (at 100, 200 and 300 K, with restraining force constants of 10,000, 5000 and $50 \hbox { kJ mol}^{-1} \hbox { nm}^{-2}$, respectively). After an additional (unrestrained) 10 ps equilibration *NVT* run, unrestrained *NpT* simulations at 1.01325 bar and 300 K are performed of few nanoseconds from which interaction energies between the ligand and its environment are obtained. The length of these *NpT* production simulations can be specified by the user during model calibration. In typical cases, 2–5 different docking poses are obtained per ligand. Starting from a given protein-ligand complex conformation, two simulations are run that start from different atomic velocities, typically leading to 4–10 protein-bound simulations per ligand when using a single protein template structure. Interaction energies over the two simulations run per starting pose are averaged [20, 21]. Because of the parallelizable nature of the setup this allows to obtain free energy predictions within few hours even on a small state-of-the-art CPU cluster (ca. 10 nodes). The ligand is also simulated in explicit solvent in absence of protein and counterions in order to evaluate ligand interaction energies for the unbound state. Full details on the employed MD settings are provided elsewhere [24].

#### 5. Postprocessing and gathering of the interaction energies

Lennard–Jones and electrostatic interaction energies between the ligand and its environment are gathered during the MD simulations. Furthermore, MD postprocessing is performed to decompose the nonbonded energy contributes of the ligand with the residues that line the binding site of the protein (for the purpose of applicability domain and reliability estimation, see below). The energies obtained are stored and used during $\Delta G_{bind}$ prediction or model calibration.

### The API

An API has been created to handle the calibration of new models or the *in silico* screening of a dataset of compounds.

Upon submission of a new calculation through the API, the interaction-energies computation task is initiated through the *Job Manager*. When this process is completed, model calibration or $\Delta G _{bind}$ prediction is executed. The API provides also access to ancillary tasks: calibration of a new model involves the definition of model parameters (including e.g. definitions of the protein binding pocket, ligand-protonation states and MD simulation time) and the preparation of specific files such as for the protein topology and the formatting of the protein structure(s) in accordance with the MD and docking packages. A set of procedures is available to facilitate these preliminary processes. Additionally, every functionality is directly accessible to the user via a web GUI, which allows a user-friendly monitoring and submission of tasks.

#### Model calibration

Calibration of a new model can be performed by submitting a training set of compounds, provided that the experimental binding free energy $\Delta G^{obs}$ toward the (off-)target is included as a common associated data field in the *SDF* file for the entire series of molecules. Calibration is performed after the computation of the ligand–protein interaction energies from the different independent simulations of the training set compounds. It involves (i) LIE model parameter fitting, and (ii) Applicability Domain (AD) definition.

**Fig. 2:**
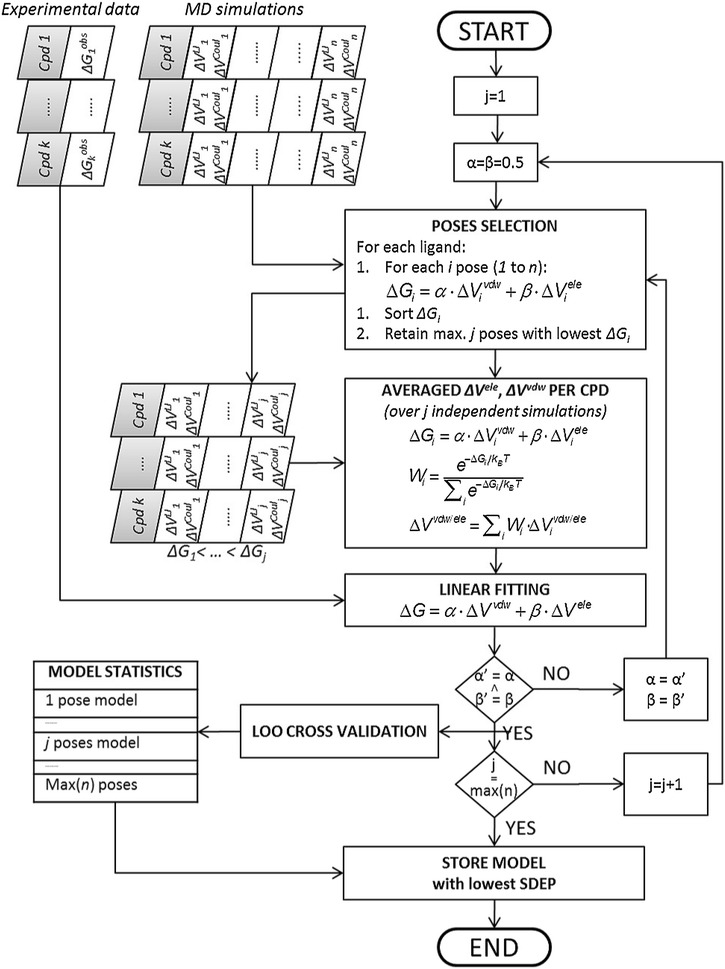
Iterative LIE fitting of a model calibrated using *k* training compounds (Cpds) for which *n* simulations are run (note that *n* can be different per Cpd). The final model will contain simulation results for the number of poses (looped over using index *j*) for which the standard deviation error in prediction (SDEP) is lowest as determined in leave-one-out (LOO) cross validation. Note that $\Delta V_{i}$’s are averaged over two MD simulations starting from different atomic starting velocities

*(i) LIE fitting* $\alpha$ and $\beta$ coefficients are fitted using an adapted version of the iterative scheme proposed by Stjernschantz and Oostenbrink [18, 24], Fig. 2. An off-set parameter $\gamma \, (\hbox {in kJ mol}^{-1}$) [42, 43] can optionally be included in model fitting, which is in that case added as a constant to Eq. (2). Initially, arbitrary values are assigned to the LIE coefficients and $\Delta G_{bind}$ is computed for every pose. The contribute of each pose to the total free energy of binding of a single compound is obtained according to Eq. (3). Using the weighted sums (according to the contribute of each pose) $\alpha$ and $\beta$ are re-optimized and the new $\alpha$ and $\beta$ coefficients are used to update the contribute of each pose to the total interaction energy for each compound, etc. [18]. This process is repeated iteratively until $\alpha$ and $\beta$ are converged.

Models including increasing number of poses with lowest $\Delta G_{bind}$ for each compound are created and evaluated based on the standard deviation error in prediction (SDEP) obtained during internal leave-one-out cross-validation. The model with lowest SDEP is stored and an applicability domain for this model is created according to the approach proposed by Capoferri et al. [24].

*(ii) AD definition* In *eTOX ALLIES*, the space of information used by the LIE model is evaluated according to five different metrics: (1) range of $\Delta G^{obs}$ values; (2) chemical similarity of the ligand, in order to take into account possible effects of rarely occurring functional groups and, implicitly, of the force-field parametrization; (3) average ligand–protein interaction energies obtained during MD simulations: to evaluate the distribution of the variables used by the model; and finally, per-residue contributes to the (4) Lennard–Jones and (5) electrostatic interactions between the ligand and protein during MD, to topographically map the interactions of the ligand with specific regions of the protein binding site (possibly characterized by different electrostatic and hydrophobic properties). During calibration of the model, the space delimited by the training set is defined as follows (cf. [24]):

1. For the range of $\Delta G^{obs}$ values, the cutoffs are defined as minimum and maximum training set values.
2. Chemical similarity is expressed as Tanimoto scores (TSs) between pairs of molecules, represented as MACCS fingerprints [44]. Every training compound is compared with the other elements of the training set and the TS with the most similar compound is stored for every ligand. The lowest value is used as cutoff.
3. To compare average ligand–protein interaction energies obtained during MD simulations, the distribution of the simulations (in terms of $\Delta V ^{LJ}$ and $\Delta V ^{Coul}$) employed during LIE fitting is characterized according to its average and covariance matrix [21].
4. Per-residue contributes to the Lennard–Jones interactions between ligand and protein: for each compound, the weighted sum (according to weights ${W_i}$ of the corresponding simulation energies) of the Lennard–Jones interaction energies is computed for every residue located in proximity of the binding site. A principal component analysis (PCA) of the residue contributes is performed, in which components are retained if they include more then 5% of the original variance, to summarize the principal ligand–protein interactions explored during fitting.
5. Per-residue contributes to the electrostatic interactions between ligand and protein: same approach as described for the analysis of Lennard Jones interactions (4).

The parameters obtained during calibration are coupled to a specific model version and can be used to estimate the reliability of the $\Delta G_{bind}$ prediction of query compounds for the specific target.

#### Prediction and reliability index

Calibrated models can be used for *in silico* screening of datasets of compounds. After gathering of the ligand–protein interaction energies, $\Delta G_{bind}$ is computed. Additionally, an index that takes into account the reliability of the prediction is provided, expressed as total number of AD metrics (0 to 5) in which the query compound is found to deviate from the training set. A query compound is considered to *not* deviate from the training set according to the different metrics reported above if [24]:

1. the predicted $\Delta G_{bind}$ value is within the range of the training set experimental values;
2. the query compound shows, for at least one training-set compound, a similarity score (as TS) that is equal or higher than the cutoff defined during calibration;
3. in terms of the average ligand–protein interaction energies, the simulations for the query compound are within 95 percentile of the training set distribution, evaluated as Mahalanobis distances from the centroid;
4. the weighted sums of the per-residue contributes to the Lennard–Jones interactions of the ligands are projected onto the principal component space of the training set and show score and orthogonal distances that are within the 95 percentile of the training set distribution;
5. the per-residue contributes to the electrostatic interactions are similar to the training set distribution when evaluated analogously as for the Lennard–Jones interactions.

A low total number of deviations (e.g. 0 or 1) corresponds to high reliability estimations, while higher numbers indicate low reliability of the predicted $\Delta G_{bind}$ [24].

#### Model preparation

Before calibration, a model needs to be configured, e.g. in terms of the choice for the protonation treatment of the ligand, by preparing the protein conformation and topology, and by defining the binding site coordinates and residues.

In *eTOX ALLIES*, model settings can be defined through the GUI, and the tedious preparation of files required for MD and docking is automated upon submission of the protein 3D structure as *PDB* format. This process is described hereafter.

1. Preparation of the protein structure: tautomeric states and rotamers of the residue side chains are obtained using *reduce* (*AmberTools*) [36].
2. Topology and coordinates of the protein are generated according to the Amber 14SB force field [45] by *LEaP* (*AmberTools*) [36]. In case the protein is a Cytochrome P450, special force-field parameters for both the heme domain and its coordinating cysteine are employed [46]. After conversion of topology and coordinate files to the *GROMACS* format by *ACPYPE* [37], hydrogen atom masses are increased to 4.032 amu to allow for timesteps of 4 fs during MD [47].
3. Structure templates for molecular docking are generated according to specific software requirements.
4. Docking software requires definition of the binding site (as radius and coordinates of the center of the sphere around it). For Cytochrome P450s, the center of docking can be automatically assigned according to the position of the heme domain atoms [21], otherwise it can be defined manually.
5. Residues lining the cavity of the binding site can be defined either manually or automatically as the residues for which any of the heavy atoms is within 16 Å from the (docking) center of the binding site.

### Extendibility

The pipeline has been developed in order to provide high flexibility in terms of exploitation of computational resources, software implementation, and job management. A list of the most important features are listed hereafter.

- High-performance computing cluster (HPCC): a specific interface is implemented that allows execution of MD simulations (the most compute-intensive part of the calculations) on a HPCC platform, instead of the local machine. Connection takes place via ssh tunnelling and is based on a *paramiko* python interface [48].
- Integration with *eTOXlab*: the *eTOXlab* software constitutes a framework for the creation of QSAR models and their deployment in production environments [49]. *eTOX ALLIES* models can be connected and used through *eTOXlab* in order to provide multiple modeling techniques in a single interface.
- Docking software: interfaces have been developed to integrate use of *PLANTS* (free for academic use) [50] and *ParaDockS* (released under GPL license) [30], which are dynamically loaded during execution of the program, according to model settings. Minor modifications to these modules can provide interfaces for other docking software packages.
- Force field: Amber-based force fields are adopted here because of the availability of free-license tools for the creation of ligand and protein topologies. All the operations that are force-field related are included in a specific module that is dynamically loaded in analogy to the docking software interfaces. Similarly, support for different force fields can be implemented in a straightforward way.
- Job identification: a specific python class has been implemented to handle submission and managing of jobs between the *API* and the *Job Manager* using *JSON* objects. In case of an extensive load of work, the class can be replaced in order to make use of a database management system.

## Results and discussion

### The web GUI

**Fig. 3:**
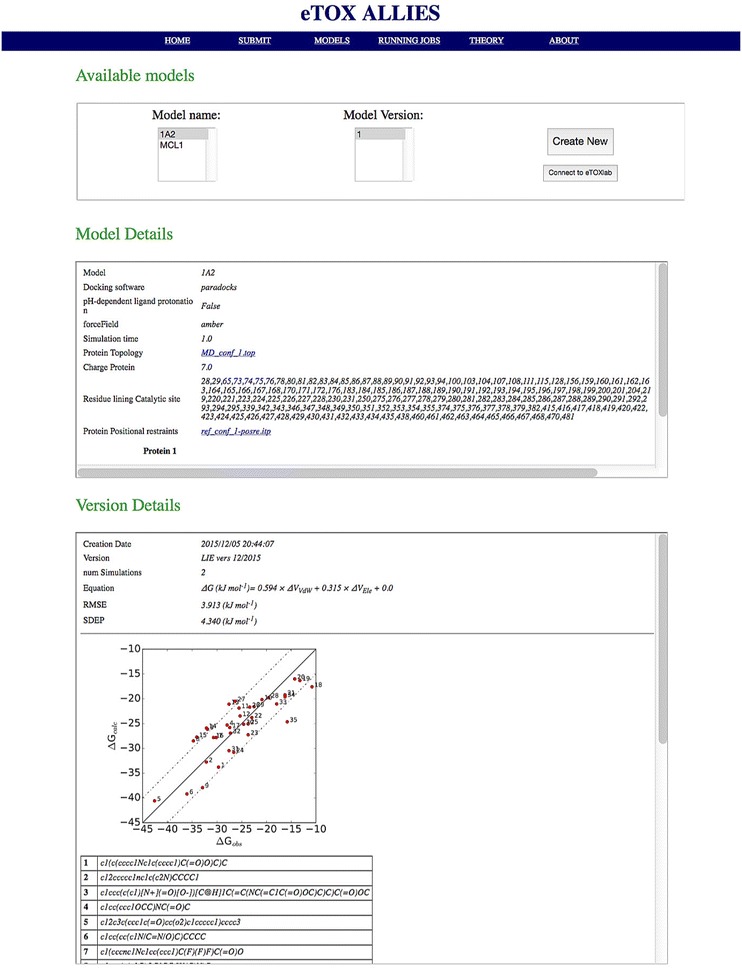
Web GUI: models page. A list of available models and calibrated versions are available here. Configuration parameters are shown in the Model Details section, while statistics about the calibrated model version are shown in the Version Details section

**Fig. 4:**
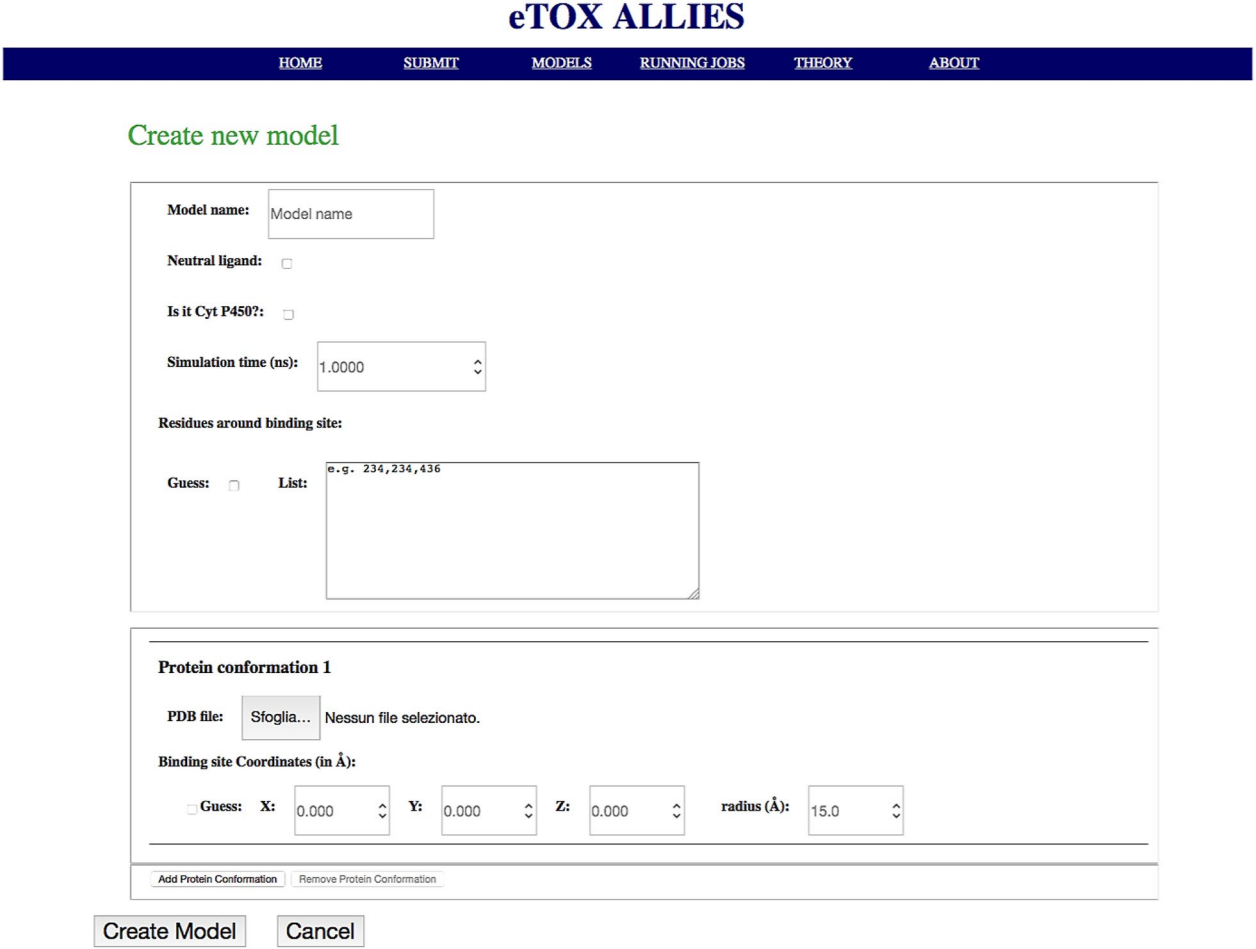
Web GUI: creating a new model. A new model can be calibrated through this page in a straightforward way. Multiple relevant protein structures can be included in a single model and uploaded in PDB format

**Fig. 5:**
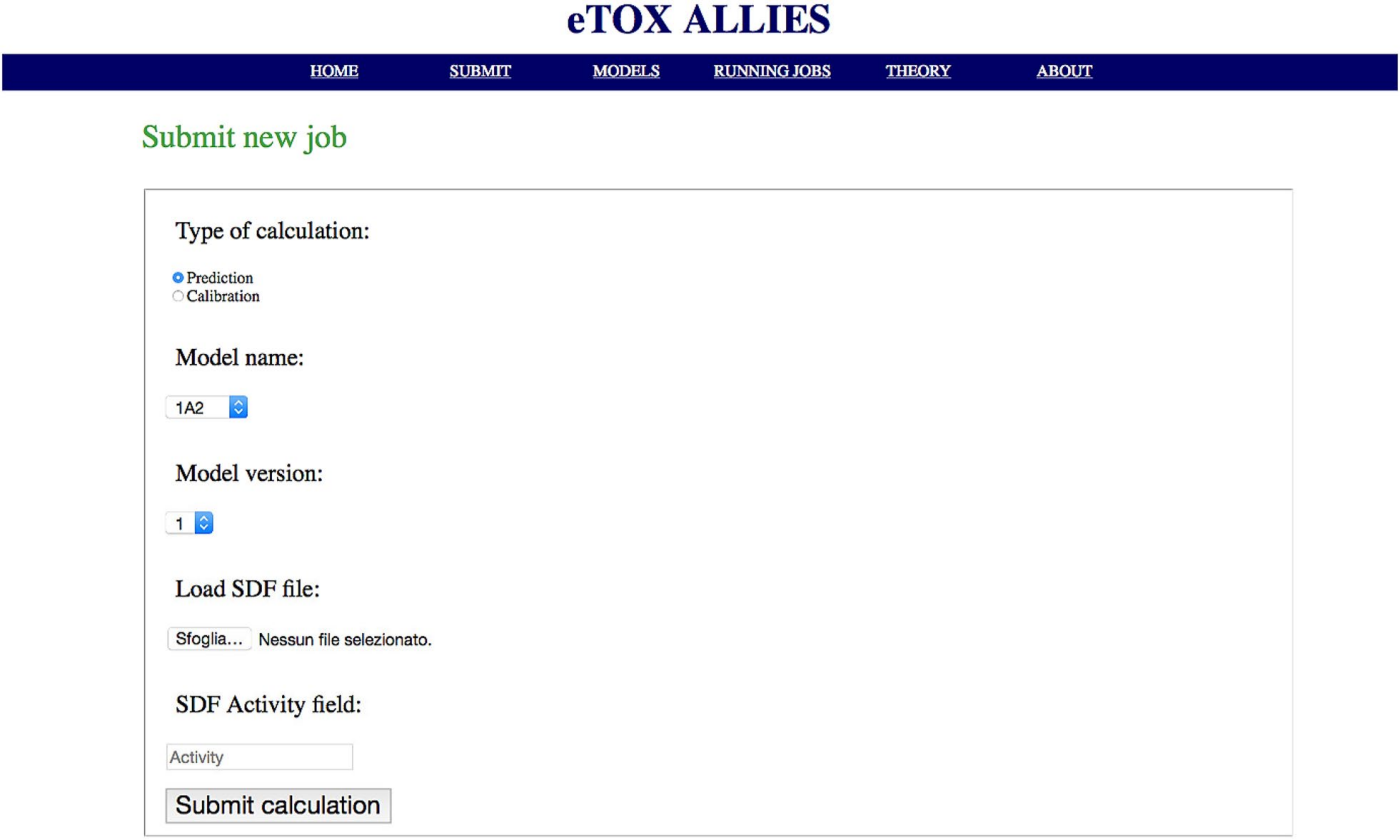
Web GUI: submit page. This page offers the possibility to submit new screenings (i.e., prediction(s) for a compound or set of compounds listed in a SDF file to be uploaded). Calibration of a new model version (recalibration) can be performed by changing the default setting for Type of calculation

**Fig. 6:**
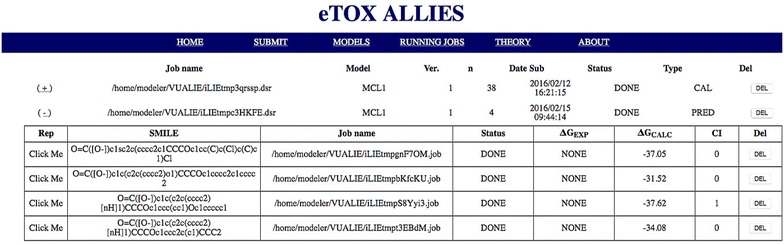
Web GUI: running jobs page Status and results from current screening(s) are shown here. For each dataset screening, a dropdown menu shows details about calculations for each compound

A web GUI has been developed that allows access to and monitoring of the functions provided by the API. The interface is directly accessible from a standard web browser, thus reducing the problem of dependency from specific libraries. In this way, the software can be deployed on a virtual machine and loaded on any machine, while being accessible from the host machine through the web browser. The functions accessible through the GUI include:

- overview of the available models, in terms of settings and statistics and creation of links with *eTOXlab* (Fig. 3);
- model preparation for a new (off-)target protein (Fig. 4);
- calibration of the model (Fig. 5);
- *in silico* screening of a dataset of compounds (Fig. 5);
- overview of the running jobs, with details about compounds, status, and results of the screening (Fig. 6).

#### Application studies

Here we demonstrate the advantage of our fully automated pipeline for (iterative) LIE model calibration and predictions for three pharmaceutically relevant proteins: Cytochrome P450 (CYP) isoform 1A2, nuclear receptor (NR) Farnesoid X receptor (FXR) and Janus Kinase 2 (JAK2). Many CYPs are flexible in nature and also NRs and kinases may bind ligands in different protein conformations and/or binding poses. Human CYPs metabolize a large variety of drug-like compounds. Drugs that tightly bind to CYPs can inhibit them and alter metabolic pathways of co-administered drugs, therefore leading to potential adverse reactions. Hence, affinity toward CYPs is of great relevance in safety pharmacology. A LIE model for binding affinity toward the isoform CYP 1A2 was recently published, in which a similar protocol as presented here was adopted [24], but in which ligand preparation was only semi-automated. Using a MD simulation time of 2.5 ns per pose, optimal $\alpha$ and $\beta$ values were found to be 0.587 and 0.267, respectively. The root mean square error (RMSE) for the model was 4.1 kJ mol^-1^ and the standard deviation error in prediction during leave-one-out cross validation (SDEP_CV_) was found to be 4.3 kJ mol^-1^. Using a different docking software (ParaDockS) than in [24], a new CYP 1A2 model was calibrated automatically using the same dataset of compounds and protein structure (for which the center of the binding site was defined automatically based on heme domain coordinates). A model was created in *eTOX ALLIES* based on simulations of 1 ns (replicated twice) for each relevant ligand binding pose, in which optimal ${\alpha }$ (0.594) and ${\beta }$ (0.315) were comparable to the published model. The RMSE was 3.9 kJ mol^-1^ and the SDEP_CV_ was 4.3 kJ mol^-1^, and the SDEP for an external set of (15) compounds for which the number of AD deviations = 0 ($\hbox {SDEP}_{EXT,0}$) was determined at 4.9 kJ mol^-1^.

As other examples, we used *eTOX ALLIES* to develop LIE binding affinity models for benzimidazole-like compounds binding to FXR in the context of the D3R Grand Challenge 2 for blind binding prediction [51, 52], and for phenylaminopyrimidines binding to JAK2. Crystal structures of the proteins were obtained from the Brookhaven protein database (PDB ID 3OMK for FXR [53], 5UT6 for JAK2 [54]) for which the center of docking was determined as the center of mass of the co-crystallized ligand (which was removed before docking). FXR model calibration and validation data were obtained for benzimidazole agonists with direct therapeutic potential and derived from $\hbox {IC}_{50}$ inhibition data reported by Richter et al. [53, 55] After splitting up these data into sets of 22 training compounds and of 8 test compounds for which all applicability domain criteria were fulfilled, the thus derived experimental binding free energies were used to obtain a LIE model (based on twice replicated 1 ns simulations) with ${\alpha }=0.333$ and ${\beta }=0.121$, and the additional off-set ${\gamma }$ with a value of $-13.0\,\hbox {kJ mol}^{-1}$ ($\hbox {RMSE}=3.8\,\hbox {kJ mol}^{-1}$, $\hbox {SDEP}_{CV}=4.1\,\hbox {kJ mol}^{-1}$, $\hbox {SDEP}_{EXT,0}=5.0\,\hbox {kJ mol}^{-1}$) [52]. For JAK2 we used two 1 ns production simulations as well and phenylaminopyrimidine $\hbox {IC}_{50}$ data from [56] for model calibration (22 training compounds; 4 test compounds with all AD criteria fulfilled). A LIE model was obtained with ${\alpha }=0.497$, ${\beta }=0.044$, $\hbox {RMSE}=4.3 \hbox { kJ mol}^{-1}$, $\hbox {SDEP}_{CV}=4.9 \hbox { kJ mol}^{-1}$ and $\hbox {SDEP}_{EXT,0}=3.8 \hbox { kJ mol}^{-1}$. Additional file 1: Figure S1 presents time series for ligand–environment interaction energies and protein-ligand atom-positional RMSDs, obtained from MD simulations used in model calibration and illustrating absence of large configurational changes, as needed when applying Eqs. (2) and (3) [19].

## Conclusions

We have presented an open source framework for unsupervised protein-ligand binding affinity (free energy) computation using iterative linear interaction energy (LIE) theory. Functionalities are available and implemented in a web GUI to submit predictions and/or (re)calibrate LIE models in a straightforward way. Output of our MD and LIE based pipeline includes predictions as well as reliability indices. External sofware only comprises open source third-party softwares, and a specific interface is implemented to enable running MD simulations on high-performance computing clusters.

## Availability and requirements

*Project name:* eTOX ALLIES

*Project home page:* https://github.com/GeerkeLab/eTOX-ALLIES

*Operating systems:* Linux OS or Mac OS X

*Programming language:* Python (MD runner in Bash)

*Other requirements:* ParaDockS or PLANTS, GROMACS 4.5.x, ACPYPE, AmberTools15

*License:* GPL v2

*Any restrictions to use by non-academics:* None.

## Additional file

**Additional file 1: Figure S1.** Time series for a selection of MD simulations used to calibrate the CYP 1A2 (left panels), FXR (middle panels) and JAK2 LIE models (right panels): Coulomb (blue) and van der Waals (violet) interaction energies (V) between the ligand and its environment (upper three rows); and protein (backbone)-ligand atom root-mean-square positional deviations (RMSD) from the MD starting structure (bottom row, different colors refer to individual RMSD time series).

## Abbreviations

AD: applicability domain
API: application programming interface
CYP: cytochrome P450
FEP: free energy perturbation
GUI: graphical user interface
HPCC: high-performance computing cluster
LIE: linear interaction energy
LOO: leave-one-out
MD: molecular dynamics
PCA: principal component analysis
QSAR: quantitative structure-activity relationship
RMSE: root mean square error
SDEP: standard deviation error in prediction
SDF: structure data file
TI: thermodynamic integration
TS: tanimoto score
