# Supplementary material for: eTOX ALLIES: an automated pipeLine for linear interaction energy-based simulations
Source: J Cheminform. 2017 Nov 21;9:58. doi: 10.1186/s13321-017-0243-x (PMC5696310; doi:10.1186/s13321-017-0243-x)
Supplement: Supplementary file 1 — Additional file 1: Figure S1. Time series for a selection of MD simulations used to calibrate the CYP 1A2 (left panels), FXR (middle panels) and JAK2 LIE models (right panels): Coulomb (blue) and van der Waals (violet) interaction energies (V) between the ligand and its environment (upper three rows); and protein (backbone)-ligand atom root-mean-square positional deviations (RMSD) from the MD starting structure (bottom row, different colors refer to individual RMSD time series). [file 13321_2017_243_MOESM1_ESM.pdf]

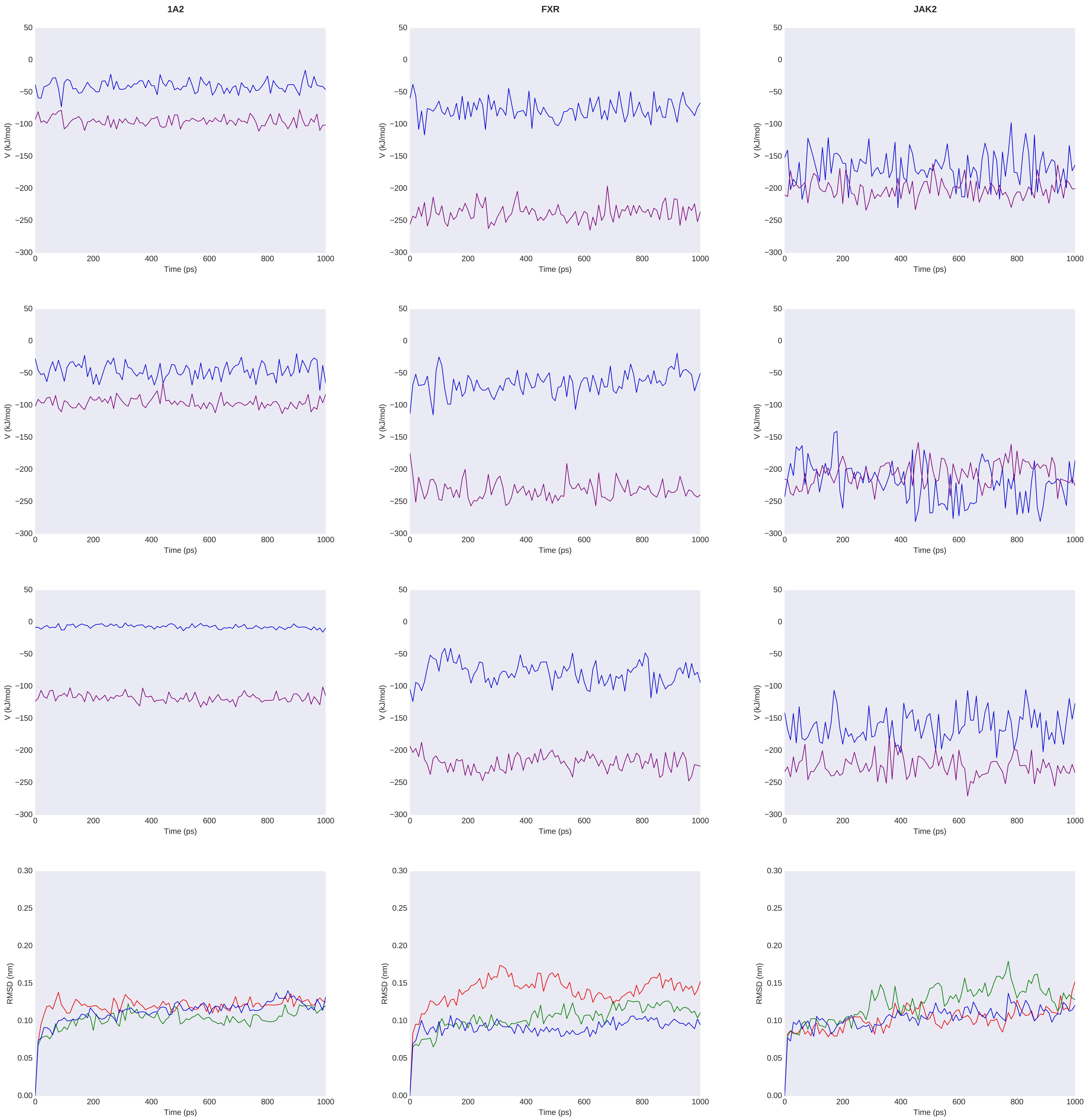
1A2
FXR
JAK2
V (kJ/mol)
RMSD (nm)
Time (ps)
50
0
−50
−100
−150
−200
−250
−300
0.30
0.25
0.20
0.15
0.10
0.05
0.00
0
200
400
600
800
1000
